# Locomotion and muscle mass measures in a murine model of collagen-induced arthritis

**DOI:** 10.1186/1471-2474-10-59

**Published:** 2009-06-03

**Authors:** Anita Hartog, Judith Hulsman, Johan Garssen

**Affiliations:** 1 Danone Research, Centre for Specialised Nutrition, Bosrandweg 20, 6704 PH Wageningen, The Netherlands; 2 Department of Pharmacology & Pathophysiology, Utrecht Institute for Pharmaceutical Sciences (UIPS), Utrecht University, Sorbonnelaan 16, 3584 CA Utrecht, The Netherlands; 3 Centre for Laboratory Animals (CKP), Wageningen University, PO Box 8129, 6700 EV Wageningen, The Netherlands

## Abstract

**Background:**

Rheumatoid arthritis (RA) is characterized by chronic poly-arthritis, synovial hyperplasia, erosive synovitis, progressive cartilage and bone destruction accompanied by a loss of body cell mass. This loss of cell mass, known as rheumatoid cachexia, predominates in the skeletal muscle and can in part be explained by a decreased physical activity. The murine collagen induced arthritis (CIA) model has been proven to be a useful model in RA research since it shares many immunological and pathological features with human RA. The present study explored the interactions between arthritis development, locomotion and muscle mass in the CIA model.

**Methods:**

CIA was induced in male DBA/1 mice. Locomotion was registered at different time points by a camera and evaluated by a computerized tracing system. Arthritis severity was detected by the traditionally used semi-quantitative clinical scores. The muscle mass of the hind-legs was detected at the end of the study by weighing. A methotrexate (MTX) intervention group was included to study the applicability of the locomotion and muscle mass for testing effectiveness of interventions in more detail.

**Results:**

There is a strong correlation between clinical arthritis and locomotion. The correlations between muscle mass and locomotion or clinical arthritis were less pronounced. MTX intervention resulted in an improvement of disease severity accompanied by an increase in locomotion and muscle mass.

**Conclusion:**

The present data demonstrate that registration of locomotion followed by a computerized evaluation of the movements is a simple non invasive quantitative method to define disease severity and evaluate effectiveness of therapeutic agents in the CIA model.

## Background

Rheumatoid arthritis (RA) is a systemic inflammatory autoimmune disorder affecting approximately 1% of the general population in the western countries. The disease is characterized by a chronic poly-arthritis, synovial hyperplasia and erosive synovitis, progressive cartilage and bone destruction and an accelerated loss of muscle mass, also known as rheumatoid cachexia [1]. The average loss of body cell mass (BCM) among patients with RA is between 13 and 15% [2]. The BMC consists primarily of muscle mass, visceral mass and immune cell mass. A decrease in muscle mass can in part be explained by a decreased physical activity [3]. This decrease in physical activity in RA patients is closely related to pain, characterized by hyperalgesia and spontaneous pain, mostly caused and exacerbated by inflammatory mediators (cytokines, prostaglandins) [4]. Other factors contributing to muscle protein wasting are increased levels of systemic and local markers of inflammation (e.g. TNF-α, IL-1β and IL-6) as well as increased levels of oxidative stress [5].

The collagen-induced arthritis model (CIA) in mice is an extensively studied RA model. It has been used to provide insight into the underlying disease process of RA and is frequently used to study the potential of new experimental therapies [6-8]. The development and severity of arthritis in the CIA model is mostly detected by a semi-quantitative clinical scoring system based on the severity of arthritis in the peripheral joints [9]. Despite of being the most widely used rodent model for RA, its use for studying arthritic pain has been reported just recently [10]. Moreover, only in small number of studies locomotion was one of the readouts in the CIA models [11,12].

The present study evaluated locomotion (changes which are at least partially pain induced), muscle mass (changes might be inflammation and locomotion induced) and clinical arthritis scores in the CIA model. The study aims to determine the applicability of locomotion and muscle mass changes as readout parameters in the CIA mouse model and its relevance for intervention studies.

## Methods

All experimental procedures using laboratory animals were approved by an independent animal experiments committee (DEC Consult, Bilthoven, The Netherlands).

### Induction of CIA

Male DBA/1 mice (Taconic, Lille Skensved, Denmark), aged 9 weeks at the start of the experiment were acclimatized in the animal housing facility starting two-weeks prior to the start of the experiment. All animals were housed in filter top cages and had free access to a water and food. The food was applied as a daily fresh prepared dough, this to simplify the food intake in the diseased state. The mice were immunized by a subcutaneous injection of 100 μg native bovine collagen type II (Chondrex, Zurich, Switzerland) emulsified in complete Freund Adjuvant (CFA, Chondrex), at the base of the tail. An intra-peritoneal booster of 100 μg of collagen type II in phosphate buffered saline (PBS) was given 21 days later. Mice with a clear onset of arthritis at day 21 were excluded from the experiment. After the booster 100% of the animals developed arthritis within 9 days. To evaluate the effect of pharmaceutical treatment on arthritis development and locomotion one group of animals was treated with Methotrexate, a frequently used disease-modifying anti-rheumatic drug (DMARD). Methotrexate (MTX, Emthexate PF, Pharmachemie B.V., Haarlem, The Neterlands) was injected three times a week (1 mg/kg, intra-peritoneal) starting at the day of the collagen booster (day 21). Control mice were injected with PBS.

### Assessment of CIA

After the booster the mice were examined three times a week for visual appearance of arthritis. Clinical severity of arthritis of the peripheral joints was graded on the level of "macroscopic" inflammation on a scale of 0 to 4 [9]. 0, no symptoms, 1 significant -, 2 moderate -, 3 marked – and 4 indicates maximal redness and swelling of the paw. The scores of all paws were summarized to obtain the "arthritis score", with a maximum of 16 for each mouse. Mice with arthritis score of 12 or higher were for ethical reasons excluded from the study. The mean arthritis score for each group was calculated (mean ± SEM). Assessment of the arthritis score was performed by two independent observers.

### Assessment of locomotion

Twice, before the arthritis induction and at 9 days after the collagen booster, mice were placed individually in an acrylic movement box of 60 × 40 cm. Spontaneous, exploratory locomotion of the animals was detected by a camera which was positioned above the "movement" boxes. The movements were registered for 5 minutes, starting 2 minutes after the mice have been placed into the boxes. The movements were evaluated by a computerized tracing system and image analyzer (EnthoVision 3.1, Noldus, Wageningen, The Netherlands). The moved distance (in cm) for each group was calculated and averaged (mean ± SEM). Changes in moving distance were calculated for each mouse as % of initial movement.

### Detection of skeletal muscle mass

12 days after the collagen booster the animals were sacrificed and the different skeletal muscles from the hind leg, tibialis anterior (TA), gastrocnemius, soleus and exterior digitorum longus (EDL) were dissected and weighted.

### Statistical analysis

Averaged values are expressed as mean ± standard error of the mean (SEM). Correlations were calculated using the Pearson's linear regression model. The changes induced by MTX were calculated by the independent-samples T-Test.

## Results

All results and conclusions are based on the data from animals with a maximum arthritis score of 12

### Arthritis development

**Figure 1:**
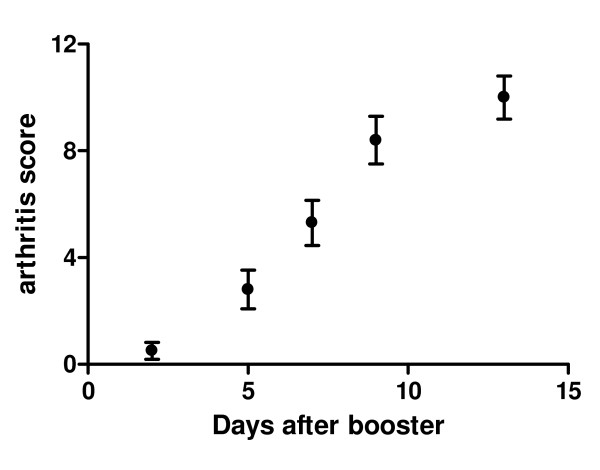
**Arthritis development, depicted as development after the collagen booster (day 21)**. Values are expressed as the mean score ± SEM, n = 17.

Arthritis development was detected in two independent experiments. Twelve animals were included in each of the test groups. At day 21, two animals from each test group developed clinical arthritis, as detected by the arthritis score, and were excluded from the rest of the experiment. After the collagen booster (day 21) the arthritis score was detected three times a week, animals with a score above 12 were sacrificed. The score of the different animals was averaged; the arthritis development was depicted in figure 1.

### Assessment of locomotion

**Figure 2:**
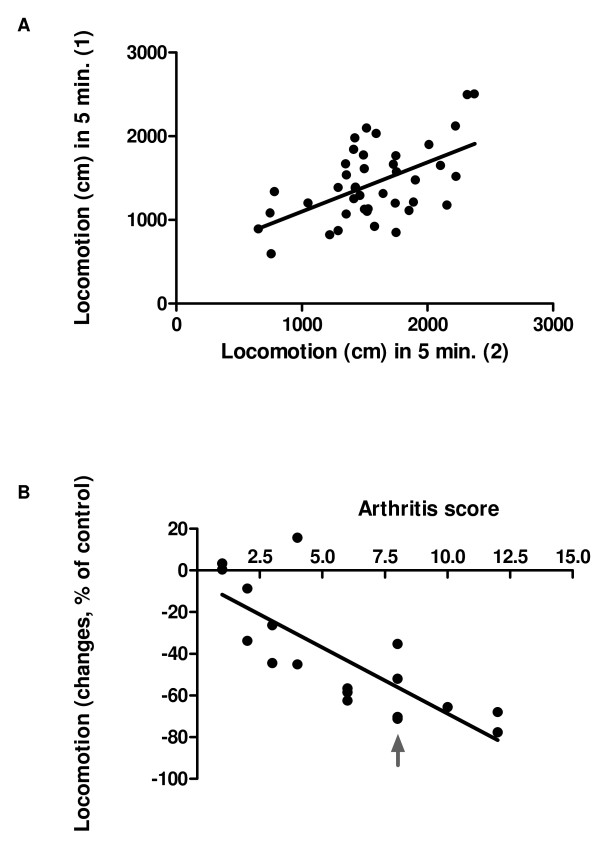
**Evaluation of locomotion in healthy and arthritis animals**. Locomotion was tested at 2 different time point (1 and 2), with a 5 days interval, in a control group of animals (A, n = 42). At day 30, 9 days after the collagen booster locomotion was tested and changes were expressed as % changes of the initial locomotion against the arthritis score of the animal (B, n = 17).

**Figure 3:**
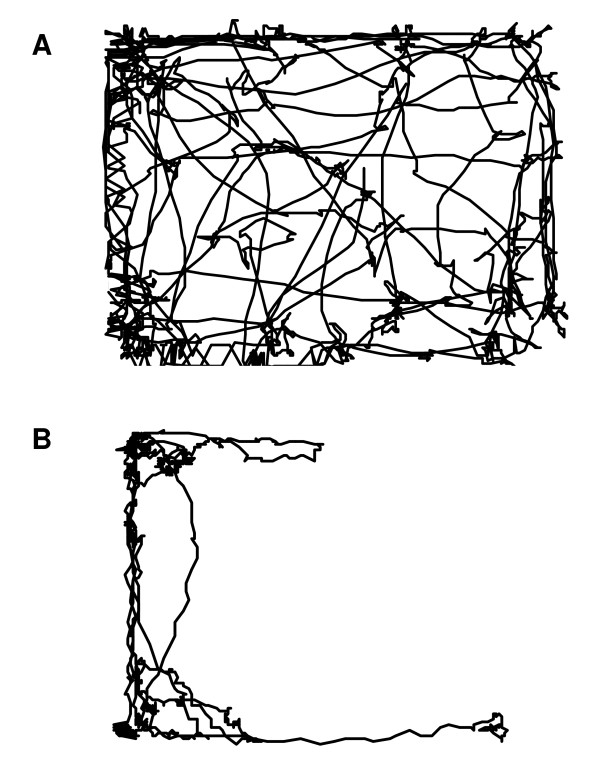
**Reproduction of the locomotion pattern before arthritis development (A) and at 9 days after the collagen booster (B), arthritis score 8**. The mouse depicted corresponds to mouse which is marked with an arrow in figure 2B.

**Figure 4:**
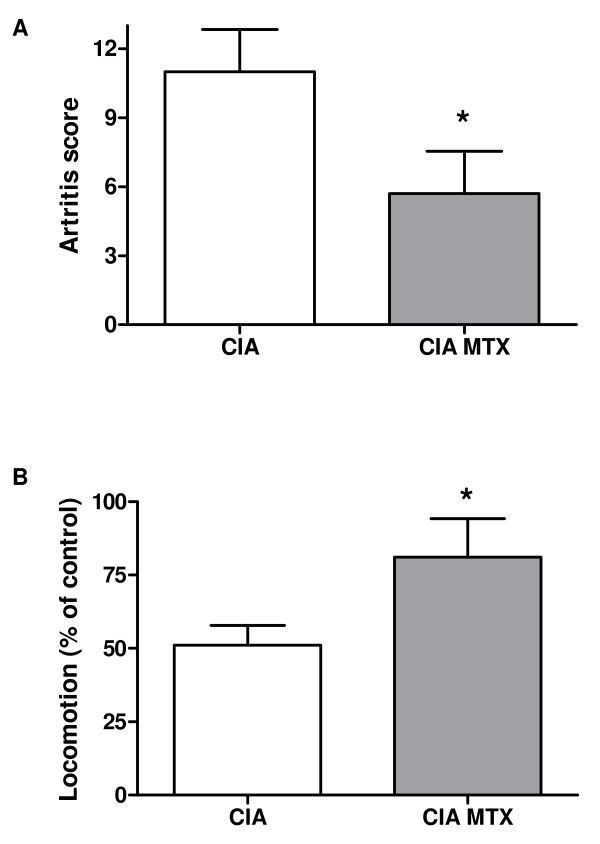
**The effects of MTX treatment on the arthritis score and locomotion, as detected nine days after the collagen booster, were depicted in figure A and B respectively**. Values are expressed as the mean ± SEM. Significant differences vs. the CIA condition were indicated by * (P < 0.05).

To investigate the reproducibility of the locomotion test, locomotion was tested twice in a control group of animals, with an interval of 5 days. The results were depicted in figure 2A. There is a significant correlation (Pearson r = 0.55, p = 0.0002) between the distance moved by the different animals at the first detection day and the distance moved at the second detection day. The presence of "lazy" and "active" animals indicates a need for individual calculation of movement changes. The average movement of each mouse as detected in two measurements before the start of arthritis induction was set at 100%. At day 30, 9 days after the collagen booster, locomotion was tested again and changes in movement, as a percentage of the initial movement, were calculated. The individual movement changes were depicted against the individual arthritis score at the detection day. There is a clear correlation (Fig. 2B) between the decrease in locomotion and the arthritis score (Pearson r = -78, p = 0.0001). The locomotion pattern of the mouse in figure 2B which is marked with an arrow was depicted in figure 3. Figure 3A shows the locomotion pattern of the mouse before arthritis initiation while figure 3B indicates the movement pattern after arthritis development (arthritis score at the time of the movement detection is 8). In order to test the relevance of the locomotion parameter for testing therapeutics, the effect of MTX treatment on the locomotion and the arthritis score were detected. At nine days after the collagen booster the MTX treatment resulted in a decreased arthritis score of 48% (Fig. 4A) and an increase of locomotion of 60% (Fig. 4B).

### Muscle mass

**Figure 5:**
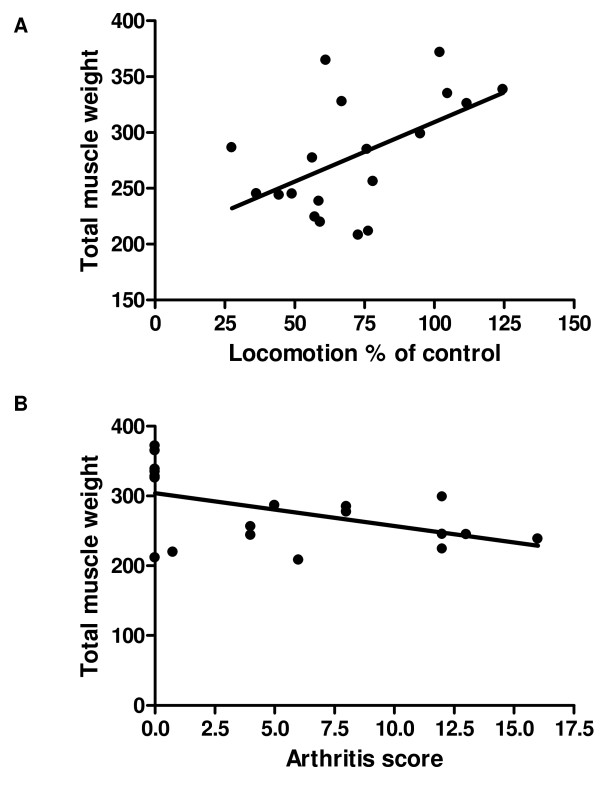
**Correlation between muscle weight and locomotion (A) and arthritis score (B)**. The total weight of the different skeletal muscles from the hind legs (tibialis anterior, gastrocnemius, soleus and exterior digitorum longus), weight 12 days after the collagen booster, was depicted as a function of the % of locomotion after arthritis development (A, n = 20) and arthritis score (B, n = 20).

**Figure 6:**
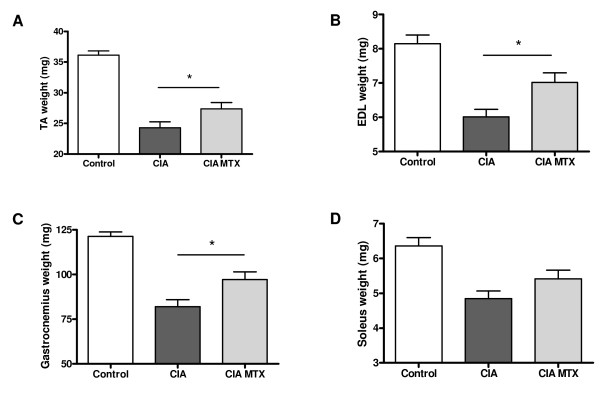
**The effect of MTX treatment on the weight of the different skeletal muscles, tibialis anterior (A), exterior digitorum longus (B), gastrocnemius (C), soleus (D) weight 12 days after the collagen booster**. Values are expressed as the mean ± SEM. Significant differences between the CIA condition and the MTX treated CIA condition were indicated by * (P < 0.05).

After the mice were sacrificed at 12 days after the collagen booster the different skeletal muscles form the hind legs were dissected and weight. The changes in total weight of the skeletal muscles of hind legs correlated significantly (Pearson r = 0.53, p = 0.02) with the % of the initial locomotion as detected 9 days after the collagen booster (final' movement study) (Fig. 5A) and with the arthritis score (Fig. 5B, Pearson r = -0.49, p = 0.03). Comparison of the individual muscles with locomotion or arthritis score did not result in a significant correlation (data not shown). MTX treatment, starting at the day of the collagen booster, was able to significantly reduce the decrease in muscle mass of the TA (Fig. 6A), the EDL (Fig. 6B) and gastrocnemius (Fig. 6C). The effect of MTX treatment on the soleus was however not significant (Fig. 6D).

## Discussion

The mouse model of CIA has been proven to be a useful animal model for RA research because it shares many immunological and pathological features with human RA [13,14]. In the present study it was demonstrated that there is a strong correlation between the macroscopic arthritis score and locomotion. This locomotion test, by which mice were placed in a new surrounding, strongly correlates with the open field-tests performed to study incidence and duration of certain behaviors [15]. Corresponding results have been indicated by Inglis et al. studying hyperalgesia [10] and Millecamps et al. studying behavior in a monoarthritic rat model [11]. However, in these studies the correlation between locomotion and the disease severity was not tested. To evaluate possible corruption of the locomotion values by habituation, the effect of repeated measurements was tested. No differences in walking distance were detected in control mice between different days of assaying (5 tests with an interval of 3–7 days between each test, data not shown). The repeated detection of locomotion in control mice did reveal however, a mouse dependent initial locomotion. These results stress the need for determination of the initial locomotion distance for each individual mouse. These initial values were set by averaging the locomotion values detected on two separate days before the start of the experiment. The present data indicate that the quantitative detection of locomotion strongly corresponds to clinical changes as detected by the semi-quantitative detection of disease severity. Also the total mass of the skeletal muscles of the hind legs, as detected at the end of the experiment, revealed to correlate to locomotion. These results suggest a direct relation between movement and muscle mass. However, movement is not the only factor effecting the muscle mass in the CIA model. In contrast to the fact that food intake by RA patients does not differ form the intake by healthy people [16,17] a strong significant decreased food intake by the CIA animals has been detected (data not shown). Moreover, TNF-α which is believed to be a central mediator of muscle wasting in RA exerts a powerful influence on muscle protein turnover resulting in a net muscle protein wasting [18,19]. Increased serum levels of TNF-α were detected in arthritic mice at the end of the experiment (data not shown). Besides, direct effects of pro-inflammatory cytokines on muscle metabolism, they play a role in hyperalgesia resulting in decreased movement. These interactions might indicate that muscle mass might be a perfect biomarker for disease severity. However, the significant decrease in food intake hampers the correlations between muscle mass loss and arthritis score although a weak correlation between total muscle mass and arthritis score could be detected.

A MTX intervention group was included to study more detailed the applicability of locomotion detection for testing treatment effectiveness of pharmaceuticals or other disease interventions. MTX is the most frequently used DMARD. Although the precise mechanisms in the treatment of RA are not completely clear, MTX exerts a variety of pharmacological actions resulting in suppression of the disease activity and reduced joint damage [20,21]. In the present CIA study the effects of MTX treatment on arthritis score, locomotion and muscle mass were studied. In agreement with previous publications [22] MTX inhibited the arthritis development in the CIA model. Moreover, treatment with MTX results in an increased locomotion. The MTX data are in agreement with the finding that the arthritis score displays an inverse correlation with locomotion. A protective effect of MTX treatment was also detected on the muscle masse of the TA, the EDL and gastrocnemius. Although the correlation between muscle masse and arthritis score was weak a clear modifying effect by MTX could be detected in the separate muscles.

## Conclusion

The present data indicate that movement detection by camera followed by a computerized evaluation of the locomotion is a simple non invasive quantitative method to follow disease development or disease modulation by interventions in the CIA model.

## Competing interests

The authors declare that they have no competing interests.

## Authors' contributions

AH and JG contributed to the study design and the manuscript preparation. AH contributed to the analysis and the interpretation of the data. JH contributed to the study design and the acquisition of the data. All authors read and approved the final manuscript.

## Pre-publication history

The pre-publication history for this paper can be accessed here:


